# Comparative gene expression profiles in pancreatic islets associated with agouti yellow mutation and PACAP overexpression in mice

**DOI:** 10.1016/j.bbrep.2015.06.006

**Published:** 2015-06-24

**Authors:** Kazuya Ikeda, Shuhei Tomimoto, Soken Tsuchiya, Ken-ichi Hamagami, Norihito Shintani, Yukihiko Sugimoto, Atsushi Ichikawa, Atsushi Kasai, Takanobu Nakazawa, Kazuki Nagayasu, Atsuko Hayata-Takano, Akemichi Baba, Hitoshi Hashimoto

**Affiliations:** a Laboratory of Molecular Neuropharmacology, Graduate School of Pharmaceutical Sciences, Osaka University, 1-6 Yamadaoka, Suita, Osaka 565-0871, Japan; b Research Fellow of the Japan Society for the Promotion of Science, Japan; c Department of Physiological Chemistry, Graduate School of Pharmaceutical Sciences, Kyoto University, 46-29 Yoshida Shimoadachi-cho, Sakyo-ku, Kyoto 606-8501, Japan; d Department of Pharmaceutical Biochemistry, Kumamoto University Graduate School of Pharmaceutical Sciences, Oe-Honmachi, Kumamoto 862-0973, Japan; e Institute for Biosciences, Mukogawa Women’s University, 11-68 Koshien-Kyubancho, Nishinomiya-shi, Hyogo 663-8179, Japan; f iPS Cell-based Research Project on Brain Neuropharmacology and Toxicology, Graduate School of Pharmaceutical Sciences, Osaka University, 1-6 Yamadaoka, Suita, Osaka 565-0871, Japan; g Molecular Research Center for Children’s Mental Development, United Graduate School of Child Development, Osaka University, Kanazawa University, Hamamatsu University School of Medicine, Chiba University and University of Fukui, 2-2 Yamadaoka, Suita, Osaka 565-0871, Japan; h Faculty of Pharmaceutical Sciences, Hyogo University of Health Science, 1-3-6 Minatojima, Chuo-ku, Kobe, Hyogo 650-8530, Japan

**Keywords:** GO, gene ontology, KK*Ay* mice, lethal yellow agouti diabetic mice, PACAP, pituitary adenylate cyclase-activating polypeptide, PACAP-Tg mice, transgenic mice overexpressing PACAP in pancreatic β-cells, PACAP, Lethal yellow agouti (KK*Ay*) diabetic mice, Gene expression, Ribosome, Mitochondrion, Chromosome

## Abstract

In diabetes mellitus, pituitary adenylate cyclase-activating polypeptide (PACAP) has insulinotropic and glucose-lowering properties. We previously demonstrated that transgenic mice overexpressing PACAP in pancreatic β-cells (PACAP-Tg) show attenuated pancreatic islet hyperplasia and hyperinsulinemia in type 2 diabetic models. To explore the underlying mechanisms, here we crossed PACAP-Tg mice with lethal yellow agouti (KK*Ay*) diabetic mice, and performed gene chip analysis of laser capture microdissected pancreatic islets from four F_1_ offspring genotypes (wild-type, PACAP-Tg, KK*Ay*, and PACAP-Tg:KK*Ay*). We identified 1371 probes with >16-fold differences between at least one pair of genotypes, and classified the probes into five clusters with characteristic expression patterns. Gene ontology enrichment analysis showed that genes involved in the terms ribosome and intracellular organelles such as ribonucleoprotein complex, mitochondrion, and chromosome organization were significantly enriched in clusters characterized by up-regulated genes in PACAP-Tg:KK*Ay* mice compared with KK*Ay* mice. These results may provide insight into the mechanisms of diabetes that accompany islet hyperplasia and amelioration by PACAP.

## Highlights

• PACAP overexpressed in KK*Ay* diabetic mice is known to exert antidiabetic effects.
• We performed gene chip analysis of pancreatic islets in these mice.
• Gene ontology analysis was performed for genes classified into five clusters.
• Genes involved in the terms ribosome, mitochondrion, and chromosome were enriched.
• These pathways may be involved in the mechanism by which PACAP ameliorates diabetes.

## 1 Introduction

Pancreatic islet β-cell mass increases in obese-diabetic rodent models including ob/ob mice [1], db/db mice [2], lethal yellow agouti (KK*Ay*) mice [3], [4], and high-fat diet-fed mice [5], compared with non-obese controls. However, islet β-cell hyperplasia is not necessarily associated with increased insulin release to compensate for increased insulin demand after overt diabetes [5], [6], [7]. Therefore, increasing β-cell function and mass, and preventing β-cell aggravation in type 2 diabetes, will greatly improve currently available therapies as well as future cell-based therapies involving pancreatic β-cell replenishment.

Pituitary adenylate cyclase-activating polypeptide (PACAP), a glucoincretin peptide member that also includes glucagon-like peptide 1, enhances glucose-induced insulin secretion and partially mediates dipeptidyl peptidase-4 inhibition-induced insulin response [8], [9], [10], [11]. We previously generated transgenic mice overexpressing PACAP in pancreatic β cells under the control of human insulin promoter (PACAP-Tg), and reported that PACAP-Tg mice show ameliorated streptozotocin-induced type 1 diabetes [12], normalized hyperplasia of pancreatic islets, and attenuated hyperinsulinemia in mouse models of type 2 diabetes such as KK*Ay* mice and high-fat diet feeding [3], [4], [13], [14]. Furthermore, we observed that attenuated islet hyperplasia in PACAP-Tg:KK*Ay* mice is due to a decrease in islet density, and not size [3].

To determine the underlying mechanisms, here we examine gene expression profiles in hyperplastic islets and those suppressed by PACAP overexpression. RNA extraction from the pancreas is challenging owing to high RNase levels and diffuse islet distribution: although islets are scattered throughout the pancreas, they occupy only a few percent area of the total organ. Thus, RNA extraction methods such as laser capture microdissection are crucial for isolating islet RNA from intact pancreatic tissue [15].

In the present study, we crossed PACAP-Tg mice with KK*Ay* mice, and performed gene chip analysis of laser capture microdissected pancreatic islets from four F_1_ offspring genotypes (wild-type, PACAP-Tg, KK*Ay*, and PACAP-Tg:KK*Ay*), coupled to Gene Ontology (GO) term enrichment analysis.

## 2 Materials and methods

### 2.1 Animals

All animal care and handling procedures were approved by the Animal Care and Use Committee of the Graduate School of Pharmaceutical Sciences, Osaka University. Generation of PACAP-Tg mice has previously been reported [12]. Male KK*Ay* mice (Japan CLEA, Tokyo, Japan) were crossed with female PACAP-Tg mice on a C57BL/6 J background to produce F_1_ offspring with four male F_1_ genotypes (wild-type, PACAP-Tg, KK*Ay*, and PACAP-Tg:KK*Ay*) on a C57BL/6 J and the original Japanese KK strain hybrid background as described previously [4]. Mice were housed in a temperature-, humidity-, and light-controlled room with a 12-h light/12-h dark cycle (light on at 8 a.m.) and allowed free access to water and food (CMF, 369 kcal/100 g, Oriental Yeast Co. Ltd., Tokyo, Japan).

### 2.2 RNA extraction from islets

Pancreata were removed from 15-week-old mice, and their sections were adhered to non-coated glass slides, fixed by treatment with 75% ethanol for 30 s at −18 °C, and then dehydrated. The PixCell IIe Laser Capture Microdissection System (Arcturus, Mountain View, CA, USA) was used to isolate 50–70 islet pieces that were laser captured to CapSur (Arcturus). Cell homogeneity was confirmed microscopically prior to processing for RNA extraction. Total RNA was subsequently extracted using the Pico Pure RNA Isolation kit (Arcturus) according to the manufacturer's protocols.

### 2.3 RNA amplification, oligonucleotide microarrays, and real-time quantitative PCR

RNA amplification and oligonucleotide microarrays were performed as described previously [16], [17], [18], but with several modifications. First-round RNA amplification was performed using the RiboAmp OA RNA Amplification kit (Arcturus) to linearly amplify anti-sense RNA, according to the manufacturer's instructions. Samples with amplified anti-sense RNA products >200 ng were then subjected to second-round amplification. cDNA was synthesized using Superscript II reverse transcriptase (Invitrogen, Carlsbad, CA, USA), and biotin-labeled anti-sense RNA prepared by the Enzo High Yield RNA Transcript Labeling kit (Enzo Diagnostics, Farmingdale, NY, USA). The resulting anti-sense RNAs were hybridized to GeneChip Murine Genome U74v.2 oligonucleotide arrays (Affymetrix, Santa Clara, CA, USA) using standard methods (*n*=1 per genotype). Image files were processed using the Microarray Analysis Suite software (Affymetrix).

Real-time quantitative PCR was performed using amplified RNA from laser-captured islet cells (*n*=3 per genotype), Superscript II reverse transcriptase (Invitrogen), random hexamers, and DyNAmo SYBR Green qPCR kit (Finnzymes, Espoo, Finland). The following primers were used for PCR: mouse *PACAP*, forward, 5′-AGA AGA CGA GGC TTA CGA CCA G-3′, reverse, 5′-TTT CTT GAC AGC CAT TTG TTT TCG G-3′; mouse *GAPDH*, forward, 5′-CTC ATG ACC ACA GTC CAT GC-3′, reverse, 5′-CAC ATT GGG GGT AGG AAC AC-3′. *GAPDH* mRNA was amplified as a control.

### 2.4 Microarray data analysis

MicroArray Suite 5.0 (MAS5; Affymetrix) was used for background correction, algorithm normalization, and intensity log transforms. The signal intensity for each probe in wild-type mice was subtracted from each intensity value in the other genotypes, then all probes with maximum minus minimum values <16-fold change were removed, resulting in selection of 1371 probes. Using the *k*-means clustering algorithm, these genes were classified into five clusters.

Functional enrichment analysis of the gene clusters was based on GO pathway annotation terms, with *P* values <0.05 considered statistically significant.

## 3 Results

### 3.1 Gene expression profiles in pancreatic islets from PACAP-Tg, KK*Ay*, and PACAP-Tg:KK*Ay* mice

We performed laser capture microdissection to extract total RNA from pancreatic islet tissue for gene expression analysis of the four mouse genotypes. We subsequently amplified the RNA by two rounds of T7 polymerase-based linear amplification, obtaining enough biotin-labeled anti-sense RNA samples to perform oligonucleotide microarray assays. To determine if the amplified RNA was suitable for microarray assays, we examined mRNA expression levels of the PACAP gene (*Adcyap1*), and observed significantly overexpressed mRNA in PACAP-Tg and PACAP-Tg:KK*Ay* mice but not wild-type or KK*Ay* mice (3.4-fold higher in PACAP-Tg:KK*Ay* vs. PACAP-Tg; wild-type and KK*Ay*, under the detection levels).

**Fig. 1:**
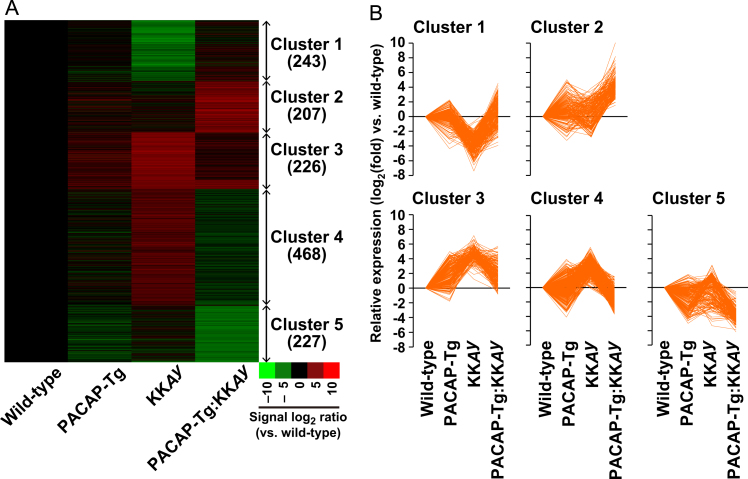
Differential gene expression in pancreatic islets from PACAP-Tg, KK*Ay*, and PACAP-Tg:KK*Ay* mice compared with wild-type mice. (A) mRNA expression levels. Colored bars represent the ratio of hybridization measurements as logarithm of fold-change vs. wild-type mice, according to the scale shown. Genes were classified into five clusters using the *k*-means clustering algorithm. (B) Gene expression patterns for clusters 1–5 in PACAP-Tg, KK*Ay*, and PACAP-Tg:KK*Ay* mice. Values represent the ratio of hybridization measurements as logarithm of fold-change vs. wild-type mice.

Among the approximately 12,000 genes represented on the oligonucleotide array, 1371 probes with hybridization signal ratios >16 (16-fold change between at least one pair of genotypes) were selected, and regarded as differentially expressed genes (Supplementary Table 1). Using the *k*-means clustering algorithm, these genes were classified into five clusters (Fig. 1A). Cluster 1 included genes with decreased expression in KK*Ay* mice, which recovered in PACAP-Tg:KK*Ay* mice. Cluster 2 included genes with slightly reduced expression in KK*Ay* mice but prominently increased expression in PACAP-Tg:KK*Ay* mice. Clusters 3–5 included genes with higher expression in KK*Ay* mice than PACAP-Tg:KK*Ay* mice, albeit at different expression levels, being higher in cluster 3 and lower in cluster 5 (Fig. 1B).

### 3.2 GO enrichment analysis of selected genes

Next, we performed GO enrichment analysis for the selected genes, and identified significantly enriched annotation terms for clusters 1 and 2 (Table 1, Table 2, Table 3). The most significantly enriched term was ribosome and its related terms, including translation and non-membrane-bounded organelle (Table 1, Table 2). The term mitochondrion and its related terms, such as mitochondrial inner membrane, were also enriched for both clusters 1 and 2 (Table 1, Table 3). The term chromosome and its related terms, including nucleosome, chromatin assembly or disassembly, chromatin organization, and chromosome organization were enriched in cluster 2 (Table 1, Table 3).

**Table 1:** GO pathway annotation terms enriched in each gene cluster.

Term	Benjamini *P* value

Cluster 1	
Ribosome	
Ribosome	2.9E−11
Structural constituent of ribosome	1.9E−08
Translation	7.2E−07
Ribonucleoprotein complex	7.3E−07
Non-membrane-bounded organelle	2.2E−06
Intracellular non-membrane-bounded organelle	2.2E−06
Mitochondrion	
Mitochondrial inner membrane	3.3E−02
Organelle inner membrane	4.4E−02
	
Cluster 2	
Ribosome	
Structural constituent of ribosome	1.7E−02
Ribosome	3.2E−02
Ribonucleoprotein complex	4.8E−02
Chromosome	
Chromosome	3.0E−02
Nucleosome	3.9E−02
Chromatin assembly or disassembly	4.3E−02
Chromatin organization	4.3E−02
Chromosome organization	4.8E−02
Mitochondrion	
Mitochondrion	4.4E−02

GO pathway annotation terms enriched in each gene cluster were determined by functional annotation analysis. *P* values represent Benjamini-corrected *P* values. After Benjamini correction, no annotation term was significantly enriched in clusters 3–5.

**Table 2:** Genes enriched for the GO term ribosome and its related terms.

Gene symbol	Gene title	PACAP-Tg	KK*Ay*	PACAP-Tg: KK*Ay*
		(log_2_(fold) vs. wild-type)		
Cluster 1				
MTAP7D1	Microtubule-associated protein 7 domain containing 1	0.7	−6.2	−1.0
CHD4	Chromodomain helicase DNA binding protein 4	0.1	−6.1	−1.2
UBXN6	UBX domain protein 6	−0.2	−5.5	−0.8
SMARCC1	SWI/SNF related, matrix associated, actin dependent regulator of chromatin, subfamily c, member 1	1.0	−5.4	−0.1
BAZ1B	Bromodomain adjacent to zinc finger domain, 1B	−0.6	−5.0	0.8
MRPS34	Mitochondrial ribosomal protein S34	0.2	−4.8	1.4
RPS19	Ribosomal protein S19	1.0	−4.6	1.5
VIL1	Villin 1	−0.3	−4.5	2.1
RPS8	Ribosomal protein S8	0.7	−4.4	2.3
H2AFV	H2A histone family, member V	0.3	−4.4	2.5
NCL	Nucleolin	−2.4	−3.9	0.8
GAR1	GAR1 ribonucleoprotein homolog (yeast)	−0.1	−3.6	3.0
NUP50	Nucleoporin 50	−2.2	−3.6	3.0
ACTR3	ARP3 actin-related protein 3	1.0	−3.5	1.8
KIF20A	Kinesin family member 20A	0.1	−3.3	1.2
PARP2	Poly (ADP-ribose) polymerase family, member 2	0.8	−3.2	2.3
EPRS	Glutamyl-prolyl-tRNA synthetase	0.4	−3.1	2.4
TUBB5	Tubulin, beta 5 class I	−0.3	−3.1	2.9
RPS10	Ribosomal protein S10	0.5	−3.1	3.6
RPS27	Ribosomal protein S27	0.6	−3.0	2.2
DYNLRB1	Dynein light chain roadblock-type 1	1.8	−3.0	1.8
FCF1	rRNA-processing protein FCF1 homolog	2.3	−2.8	3.3
DYNLL1	Dynein light chain LC8-type 1	0.6	−2.8	3.4
RPL10	Ribosomal protein L10	−0.3	−2.7	2.5
RPS26	Ribosomal protein S26	0.1	−2.7	2.9
EEF1A1	Eukaryotic translation elongation factor 1 alpha 1	−0.5	−2.5	3.0
				
Cluster 2				
RPL7	Ribosomal protein L7	0.5	−2.8	4.6
RPS3A	Ribosomal protein S3A	1.1	−2.6	4.0
RPLP0	Ribosomal protein, large, P0	1.0	−2.6	4.2
UBB	Ubiquitin B	1.4	−1.9	3.2
SNRPE	Small nuclear ribonucleoprotein E	0.8	−1.9	3.8
RPS18	Ribosomal protein S18	0.9	−1.4	4.2
RPS4X	Ribosomal protein S4, X-linked	1.0	−1.0	4.1
HNRNPA2B1	Heterogeneous nuclear ribonucleoprotein A2/B1	−1.3	−0.1	4.6
MRPL35	Mitochondrial ribosomal protein L35	1.3	0.4	5.1

Genes enriched for the GO pathway annotation terms, ribosome and its related terms (translation, ribonucleoprotein complex, and non-membrane-bounded organelle). Expression levels are indicated as logarithm of fold-change vs. wild-type mice. Representative genes with the largest changes are shown.

**Table 3:** Genes enriched for the GO terms mitochondrion, chromosome, and their related terms.

Gene symbol	Gene title	PACAP-Tg	KK*Ay*	PACAP-Tg: KK*Ay*
		(log_2_(fold) vs. wild-type)		
*Mitochondrion and related terms*				
Cluster 1				
COX6A1	Cytochrome c oxidase, subunit VI a, polypeptide 1	1.0	−4.7	3.4
MCART1	Mitochondrial carrier triple repeat 1	−0.1	−4.6	0.9
TIMM8A1	translocase of inner mitochondrial membrane 8 homolog a1 (yeast)	0.4	−3.3	1.3
SLC25A39	Solute carrier family 25, member 39	0.4	−3.1	1.4
MDH2	Malate dehydrogenase 2, NAD (mitochondrial)	−0.1	−2.7	2.0
*Cluster 2*				
ACAA2	Acetyl-Coenzyme A acyltransferase 2 (mitochondrial 3-oxoacyl-coenzyme A thiolase)	1.1	−2.4	3.9
PARK7	Parkinson disease (autosomal recessive, early onset) 7	1.8	−2.3	2.9
ALDH9A1	Aldehyde dehydrogenase 9, subfamily A1	2.2	−1.7	4.2
TOMM20	Translocase of outer mitochondrial membrane 20 homolog (yeast)	−1.1	−1.2	3.1
NDUFB5	NADH dehydrogenase (ubiquinone) 1 beta subcomplex, 5	1.7	−0.7	6.0
ACAA1B	Acetyl-coenzyme A acyltransferase 1B	0.0	−0.3	4.3
COX7C	Cytochrome c oxidase subunit 7C, mitochondrial-like	0.7	0.2	4.8
				
*Chromosome and related terms*				
Cluster 2				
SMC4	structural maintenance of chromosomes 4	0.7	−1.5	3.6
HIST1H2AB	histone H2A type 1-like	1.6	−1.5	3.1
RBM14	RNA binding motif protein 14	−0.4	−1.4	3.0
CREBBP	CREB binding protein	2.4	−0.7	3.5
KLF1	Kruppel-like factor 1 (erythroid)	0.0	−0.6	3.5
H3F3A	H3 histone, family 3A	−1.8	−0.5	2.8
MKI67	antigen identified by monoclonal antibody Ki 67	2.6	0.5	6.8
H2AFZ	H2A histone family, member Z	1.9	0.8	4.1
TOP1	topoisomerase (DNA) I	2.1	1.4	4.9
PCNA	proliferating cell nuclear antigen	0.6	2.4	5.9

Genes enriched for the GO pathway annotation terms, mitochondrion and organelle inner membrane, and chromosome, nucleosome, chromatin assembly or disassembly, chromatin organization, and chromosome organization. Expression levels are indicated as logarithm of fold-change vs. wild-type mice. Representative genes with the largest changes are shown.

## 4 Discussion

PACAP enhances glucose-induced insulin secretion and partially mediates dipeptidyl peptidase-4 inhibition-induced insulin response [8], [9], [10], [11]. Moreover, the longitudinal effect of PACAP on islets, including regulation of β-cell mass and function, have been addressed. Our previously generated PACAP-Tg mice show ameliorated streptozotocin-induced type 1 diabetes [12], normalized hyperplasia of pancreatic islets, and attenuated hyperinsulinemia in mouse models of type 2 diabetes [3], [4], [13], [14]. In ob/ob and db/db mice [2], total islet volume increases were due to an increase in mean islet mass, but not number [1], [2], while we observed that attenuated islet hyperplasia in PACAP-Tg:KK*Ay* mice is due to a decrease in the density of both hypertrophied islets and very small islets [3], [4]. Currently available medications aim to decrease glucose levels by improving insulin secretion in remaining β cells, improving insulin activity in target tissues, or replacing missing insulin [7], [19], [20]. Thus, increasing β-cell function and mass, and preventing β-cell aggravation in type 2 diabetes will greatly improve currently available therapies. In the present study, we performed comparative gene expression analysis in four mouse genotypes: wild-type and PACAP-Tg with or without KK*Ay*. Because of high RNase quantities, the diffuse distribution of pancreatic islets, and possible alteration of gene expression during collagenase-based procedures for islet isolation, we collected islet tissue from the pancreas using laser capture microdissection, which is crucial for isolating islet RNA from intact pancreatic tissue [15]. The obtained total RNA was successfully amplified, labeled, and subjected to oligonucleotide microarray assays.

Real-time quantitative PCR revealed overexpression of *PACAP* mRNA in pancreatic islets in PACAP-Tg mice and 3.4-fold higher levels of *PACAP* mRNA in PACAP-Tg:KK*Ay* mice as compared with PACAP-Tg mice. We previously showed that plasma insulin levels were significantly elevated in PACAP-Tg:KK*Ay* mice compared with PACAP-Tg mice (at 17 weeks of age, PACAP-Tg:KK*Ay* mice, 92±16 ng/ml; PACAP-Tg mice, 3.38±0.74 ng/ml) [4], suggesting a significant activation of the insulin promoter in PACAP-Tg:KK*Ay* mice. This may be a reason for the enhanced overexpression of *PACAP* mRNA in PACAP-Tg:KK*Ay* mice as compared with PACAP-Tg mice because PACAP is overexpressed under the control of the β-cell-specific insulin promoter in these mice.

Under the present experimental condition, *PACAP* mRNA levels were below the detection levels in wild-type and KK*Ay* mice, however, this does not show that PACAP is not expressed in pancreatic islets in these mice. Previously, we observed slight PACAP immunoreactivity in pancreatic islets of wild-type mice, whereas an intense immunoreactivity was observed in PACAP-Tg mice [12]. In addition, there are several reports that demonstrate the presence of PACAP in pancreatic islets [8], [9].

In the present microarray analysis, we set a threshold for differential expression at 16-fold. This threshold point is high, but reflects the high degree of expression variance among the genotypes, which may depend on almost homogenous islet tissue collection by laser capture microdissection and a near-linear RNA amplification protocol. There is a limitation to our present study: we analyzed microarray data from a single mouse per genotype. To overcome this, we set the differential expression threshold at a relatively high point but it is possible that the data are biased because of the genetic background of each mouse, and thus genes with more subtle differential expression changes may not have been identified. Another limitation is that we isolated RNA from pancreatic islets which consist of several endocrine cells including not only β cells but also α cells, δ cells and others. Although we previously showed that insulin-positive and glucagon-positive areas and the percentage of infiltrated islets were not significantly different between PACAP-Tg:KK*Ay* and KK*Ay* mice [3], we can not exclude the possibility that the observed changes of gene expression are also reflected by changes in the proportion of the cell types.

GO enrichment analysis of the genes classified in the five clusters showed significantly enriched terms for clusters 1 and 2 only. These two clusters contain genes with significantly increased expression in PACAP-Tg:KK*Ay* mice compared with KK*Ay* mice, while clusters 3–5 contained genes with the opposite changes: decreased expression in PACAP-Tg:KK*Ay* mice compared with KK*Ay* mice. For clusters 1 and 2, genes in the annotated terms, ribosome and mitochondrion and their related terms were significantly enriched. This may suggest that ribosomal-dependent translation and mitochondrion-dependent cellular processes are involved in PACAP transgene-dependent amelioration of KK*Ay* diabetic islet phenotypes [3], [4], [13]. In addition, genes involved in the annotated term chromosome and its related terms were enriched in cluster 2. As these genes were specifically up-regulated in PACAP-Tg:KK*Ay* mice, it may be possible that the effects of PACAP overexpression on KK*Ay* mice, such as attenuation of hyperplasia and hypertrophy in KK*Ay* islets [3], [4], are mediated at least partially by gene transcriptional control.

Gene transcriptome analyses have been performed in whole pancreatic islets, cultured β-cell lines, and isolated β cells by flow cytometry-based sorting using real-time PCR, microarrays, and more recently RNA sequencing analysis [21]. In addition, genome-wide association studies have identified over 60 loci associated with type 2 diabetes [22]. Nevertheless, causal variants for diabetes pathogenesis remain largely unknown, and therefore a “missing heritability” problem remains.

In conclusion, our present observations suggest that mechanisms involving ribosomal and chromatin organization, and mitochondrial function are relevant to the pathomechanism of islet hyperplasia and PACAP amelioration, and may provide insight into the preservation of β-cell function during type 2 diabetes development.

## Appendix B Transparency material

Transperancy material

Transparency material

Transparency material

Transparency material

Transparency material

Transparency material

Transparency material

Transparency material

Transparency material

Transparency material

Transparency material

Transparency material

Transparency material
